# In silico neoantigen screening and HLA multimer-based validation identify immunogenic neopeptide in multifocal lung adenocarcinoma

**DOI:** 10.3389/fimmu.2024.1456209

**Published:** 2024-12-10

**Authors:** Xin Wang, Lang Jiang, Juan Zhao, Mi Wu, Jin Xiong, Xiongwen Wu, Xiufang Weng

**Affiliations:** ^1^ Department of Immunology, School of Basic Medicine, Tongji Medical College, Huazhong University of Science and Technology, Wuhan, China; ^2^ Department of Blood Transfusion, Tongji Hospital, Tongji Medical College, Huazhong University of Science and Technology, Wuhan, China

**Keywords:** neopeptide screening, multifocal lung adenocarcinoma, NANOGNB, immunogenicity neopeptide, artificial antigen-presenting cells

## Abstract

**Background:**

Mutations commonly occur in cancer cells, arising neoantigen as potential targets for personalized immunotherapy of lung adenocarcinoma (LUAD). However, the substantial heterogeneity observed among individuals and distinct foci within the same patient presents significant challenges in formulating immunotherapy strategies. The aim of the work is to characterize the mutation pattern and identify neopeptides across different patients and diverse foci within the same patients with LUAD.

**Methods:**

Seven lung adenocarcinoma samples and matched tissues/blood are collected from 4 patients with LUAD for whole exome sequencing, mutation signature analysis, HLA binding prediction and neoantigen screening. Dimeric HLA-A2 molecules were prepared by Bac-to-Bac baculovirus expression system to establish a T cell stimulation system based on HLA-A2-coated artificial antigen-presenting cells for the validation of immunogenic neopeptides.

**Results:**

Similar mutation pattern with predominant missense mutation and high tumor mutation burden was observed across individuals with lung adenocarcinomas and between non-invasive and invasive foci. We screened and identified 3 consistent mutated genes among 100 top genes with highest mutation scores contributed across 4 patients, and 3 mutated peptides among 30 with highest HLA-A2 binding affinity distributed in at least 2 out of 4 foci in the same patient. Notably, LUAD-7-MT peptide encoded by NANOGNB demonstrated higher immunogenicity in promoting CD8+ T cells proliferation and IFN-γ secretion than the corresponding wildtype peptide.

**Conclusions:**

This study provides an in-depth analysis of mutation characteristics of LUAD and establishes a neoantigen screening and validation system for identifying immunogenicity neopeptide across individual patients and diverse foci in the same patient with multifocal LUAD.

## 1 Introduction

Lung cancer remains the leading cause of cancer incidence and mortality worldwide, signifying a critical global health challenge (1). Non-small cell lung cancer (NSCLC) is the most prevalent type of lung cancer, account for approximately 80% to 85% of all cases, with lung adenocarcinoma (LUAD) constituting over 50% of these cases (2). Advanced lung cancer is primarily treated with comprehensive therapy based on chemotherapy, along with traditional methods such as surgery and radiation therapy (3). However, the suboptimal outcomes associated with traditional approaches underscore the imperative for a paradigm shift toward targeted therapies and immunotherapies (4, 5). Tumor immunotherapies includes various approaches such as immune modulation, adoptive immunotherapy, immune checkpoint blockade, neoantigen vaccination, and more (6, 7). These approaches harness the body’s immune system and target specifically against tumors, offering a more personalized approach. However, the highly diverse genetic landscape of LUAD across individuals, as well as the notable heterogeneity among different foci within the same patient with multifocal LUAD, pose significant challenges in formulating personalized immunotherapy strategies (8, 9).

Tumor antigens can be divided into two categories: tumor-associated antigens and tumor-specific antigens (10). Tumor-associated antigens are antigens that exist in normal cells or tissues but are ectopically expressed or abnormally increased in tumor tissues. Current immune therapeutic methods targeting these antigens face challenges in inducing effective anti-tumor immune responses due to peripheral and central tolerance mechanisms (11, 12). Furthermore, as these antigens are not limited to tumor cells, their presence in normal tissues raises the danger of off-target effects. Tumor mutations give rise to novel markers that are specifically expressed in tumor tissue, making them targets for immune system. Tumor mutational burden (TMB) and tumor neoantigen burden (TNB) are measures of overall mutational burden and the neoantigen content in a tumor, respectively. Both are valuable in predicting responses to immune checkpoint blockade (13). Tumor neoantigens are novel proteins generated through somatic mutations during tumor development and progression. These proteins are specifically expressed on tumor tissue cells but not on normal tissues and cells, facilitating them specific targets for tumor immunotherapy (14). Incorporating neoantigen-based strategies in the management of LUAD holds promise for personalized and targeted immunotherapeutic approaches.

Of note, not all generated peptides qualify as tumor neoantigens, and only those capable of successfully triggering T cell responses are considered tumor neoantigens (15). At present, the predominant neoantigen identification method is to discover the site of non-synonymous mutations via tumor exome sequencing (16), which requires identifying tumor somatic mutation genes by comparing the exome sequences of tumor tissues and normal tissues and predicting the affinity of tumor mutation peptides (17). However, genomic complexity, tumor heterogeneity and validation challenges pose difficulties in accurately detecting neoantigens in LUAD, especially in cases with multifocality.

In this study, we initially investigate the mutation characteristics of lung adenocarcinoma through whole exome analysis, evaluating the overall mutation patterns of non-invasive and invasive foci in different patients and a patients with multiple foci. Notably, we develop a neoantigen screening and validation system to pinpoint the neopeptides among LUADs, aiming to lay the framework for personalized precision therapy options suited to each patient.

## 2 Materials and methods

### 2.1 Sample collection

This study included three pairs of invasive tumor tissues and corresponding adjacent non-tumor tissues from three LUAD patients (patient 1- patient 3). Additionally, four tumor tissues, including two pre-invasive foci and two invasive foci, from patient 4 with multifocal carcinomas were collected to compare with corresponding peripheral blood. All samples were obtained from Union Hospital in Wuhan, China. Patient characteristics were available in **Table 1**. The study received approval from the ethics committee of Tongji Medical College, Huazhong University of Science and Technology (2023S063), and all patients gave informed consent for research testing.

**Table 1:** Characteristics of tissues enrolled in this study.

Patient ID	Sample ID	Diagnosis	HLA-A	Diagnosis
patient 1	P1	Invasive	02:07, 02:03	LUAD^a^
patient 2	P2	Invasive	33:03, 24:02	
patient 3	P3	Invasive	02:07, 11:01	
patient 4	P4.1	Invasive	02:01, 33:03	
	P4.2	Pre-invasive		
	P4.3	Invasive		
	P4.4	Pre-invasive		

a LUAD, Lung adenocarcinoma.

### 2.2 Whole exome sequencing

In this study, we isolated DNA from blood and tissue samples taken from four LUAD patients. DNA extraction was performed using the FastPure DNA Isolation Mini Kit (Vazyme Biotech Co.,Ltd). The qualified DNA with minimal RNA contaminant and concentration exceeding 20 ng/μL evaluated by 1% agarose gel electrophoresis and Qubit 2.0 (Invitrogen) was sheared to 180 ~ 280 bp fragments with M220 Focused-ultrasonicator (Covaris). The Agilent SureSelect Human All Exon V5/V6 Kit (Agilent) facilitated library preparation and capture experiments. DNA libraries were generated by ligating adapters to both ends of the fragments following end repair, phosphorylation, and A-tailing. Subsequently, the library, tagged with a specific index, underwent liquid-phase hybridization with a biotin-conjugated probe. Exons were captured using streptomycin-coated magnetic beads followed by PCR amplification. Quality control of the DNA libraries was performed gel electrophoresis and High Sensitivity DNA assay with the 2100 Bioanalyzer System (Agilent). All samples were processed for massive sequencing in the paired-end 2 × 150 bp mode on the platform Illumina HiSeq 2500 (Illumina).

### 2.3 Genomic analysis

CASAVA 1.8 (Illumina) was utilized for image analysis and base calling to obtain the raw sequencing data, which was deposited in fastq format. The quality of the obtained fastq files was analyzed using FastQC package. Reads were aligned to UCSC hg19 (GRCh37) with BWA algorithm (18) and Samblaster algorithm (19), followed by deduplication with Samblaster algorithm. Next, Mutect2 (20) was used to identify the mutations of SNV and InDel, and the significance of gene mutation was visualized by maftools (21) and ggplot2. The WES average sequencing depth of tumor tissues and matched adjacent tissues was 150X.

### 2.4 Expression landscape and prognostic value of mutated gene set

We utilized multiple bioinformatic databases to investigate the expression and prognostic value of mutated gene set. The prognostic value of 98 genes, which are available in TCGA datasets, of top 100 mutated genes was evaluated using the (http://gepia2.cancer-pku.cn/#index) (22). The genes expression of the candidates and the pathological stage plot of NANOGNB expression were also accessed from GEPIA2.0 database. The protein expression ratio was approachable in HPA database (http://proteinatlas.org/). IHC images were obtained from the HPA database.

### 2.5 HLA typing and neoantigen prediction

HLA genotyping utilizing normal DNA fastq files and the ATHLATES algorithm (23), which takes locus-specific reads from the initial read mapping to all known HLA alleles in the IMGT/HLA database as input, for patients in our study. The Immune Epitope Database analysis resource NetMHCpan was used to predict MHC class I binding of 8 ~ 11 mer mutant peptides to the patients’ HLA-A, -B, and -C alleles (24). Peptides with IC50 mutant <500 nM and IC50 mutant < IC50 wild were considered high binders and regarded as neopeptide candidates. For the second screening, NetMHCpan (25, 26), IEDB (27, 28) and SYFPEITHI (29) algorithms were all utilized to predict binding affinity between candidates and HLA-A*0201. The pre-score (a) obtained from NetMHCpan and IEDB BA was the binding concentration of peptide candidates. The higher pre-score indicated the lower binding affinity of peptide candidates. Therefore, we transformed pre-score to be score (b) by ( $b=\frac{1}{\log a}$ ) to positively ref lect binding affinity. Additionally, fold change (FC) were calculating by divided pre-score of wild type peptides by pre-score of mutated peptides originated from NetMHCpan, reflecting affinity changes of mutated peptides versus wild-type peptides. No pre-treatment was required for the scores (b) obtained from IEDB EL and SYFPEITHI algorithms. The relative scores (c) were then given by $c=\frac{\left(b-\text{min}\;(b)\right)}{\left(\max\left(b\right)-\text{min}\;(b)\right)}\times 19+1\;\;\text{or}\;\;c=\frac{\left(FC-\text{min}\;(FC)\right)}{\left(\max\left(FC\right)-\text{min}\;\left(FC\right)\right)}\times 19+1$ to maintain a range of 1-20 for comparison and graphing.

### 2.6 Recombinant virus generation and HLA-A2-3×flag protein expression

The β2m encoding gene fragment was PCR amplified from human PBMC, and the HLA-A2-3×flag-tag fusion gene fragment was chemically synthesized. These two fragments above were cloned into the pFastBacDual baculovirus expression vector to obtained recombinant pFastBacDual-β2m-HLA-A2-3×flag-tag plasmid. Recombinant vector was transfected into DH10Bac (Invitrogen) for the generation of bacmid DNA, which was then transfected into spodoptera frugiperda (Sf9) insect cells, after all the fragments and bacmid were identified by agarose gel electrophoresis. Recombinant HLA-A2-3×flag baculovirus was then generated after three passages and the protein expression level was determined by ELISA, which utilizing W6/32 antibody (Thermo Scientific) as the coating antibody and HRP-β2m antibody (santa cruz biotechnology) as the secondary antibody.

### 2.7 aAPCs Preparation and *in vitro* neoantigen screening

The sulfate latex beads 8% (wt/vol), 3.5 μm (Thermo Fisher) were coated with purified anti-human CD28 (BD Pharmingen), anti-flag antibody (absin) followed by HLA-A2-3×flag protein to prepare HLA-A2 artificial antigen-presenting cells (aAPCs). Bovine serum albumin (BSA) was added to bind non-specific sites.

PBLs, separated from PBMC samples of six HLA-A2(+) donors, were cultured in 10% FBS-RPMI 1640 and stimulated with aAPCs at a ratio of 1:1 with the addition of 20 IU/ml recombinant IL-2 and 40 μg/ml peptides after stained with 5-(and 6)-carboxyfluorescein diacetate succinimidyl ester (CFSE) (Biolegend). Cells and supernatant were collected on the 10th days of co-culture for flow cytometry and cytometric bead array (CBA) (BD Biosciences) respectively.

### 2.8 Flow cytometry

Cells and aAPCs were surface stained with corresponding fluorescence-conjugated antibodies for 30minutes at 4°C. CFSE Data were collected using FACSVerse (BD Biosciences) and analyzed by FlowJo software (Tree Star). The fluorescence-conjugated antibodies were as follows: HLA-A2 (Biolegend), IgG (Biolegend), CD3 (Biolegend), CD8 (Biolegend).

### 2.9 Statistics analysis

R programming language (Version 4.0.4) and GraphPad Prism 8.0 were utilized for statistical analysis. Data were analyzed by Student’s t-test or ANOVA for comparisons between groups. A p-value lower than 0.05 was considered statistically significant.

## 3 Results

### 3.1 The somatic mutation profile of lung adenocarcinoma maintains a similar pattern across individuals and between invasive and non-invasive foci

**Figure 1:**
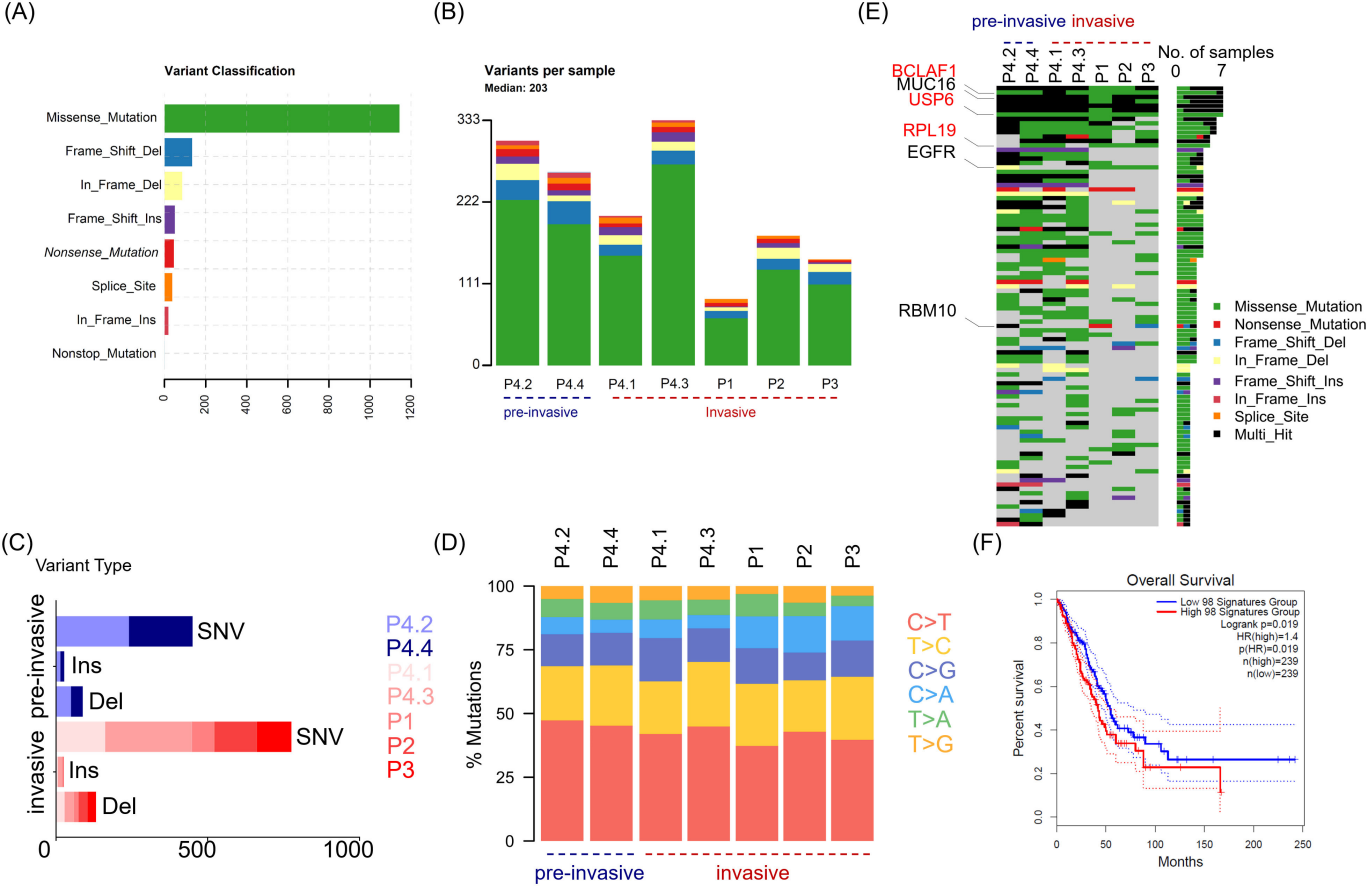
The distinguishing features of somatic mutations in LUAD across different patients and between non-invasive and invasive foci. Tumor tissues (n = 7) were collected from four patients (P1, P2, P3 and P4) with lung adenocarcinoma, and P4 with multifocal LUAD contributed four tumor tissues, including two pre-invasive foci (P4.2 and P4.4) and two invasive foci (P4.1 and P4.3). Whole exome sequencing (WES) was performed to determined somatic mutation of tumor tissue from paired adjacent non-tumor tissues or paired blood. **(A)** Bar graph depicts the frequency of indicated variants of enrolled tumor tissues, with colors showing different variant classifications. **(B)** Stacked bar plot shows the frequency of variants in individual tumor tissues. The tissues are divided into two group including pre-invasive group (P4.2, P4.4) and invasive group (P4.1, P4.3, P1, P2, P3). **(C)** Stacked bar plot shows the frequency of single nucleotide variants (SNV), inserts (Ins) and deletions (Del), in indicated tissues. **(D)** Scaled stacked bar plot depicts ratios of six kinds of SNV mutations in indicated tissues. **(E)** The top 100 genes with the highest mutation rates were displayed in descending order of mutation frequency. **(F)** Survival chart depicts overall survival rates in LUAD patients with high (red) and low (blue) transcriptomic signatures of 98 genes of top 100 mutated genes, which are available in TCGA datasets. Colors showing different variant classifications in **(A, B, E)**: Green (Missense mutation), Blue (Frame shift deletion), Yellow (In frame deletion), Purple (Frame shift insertion), Red (Nonsense mutation), Orange (Splice site mutation), Amaranth (In frame insertion), Azure (Nonstop mutation).

In order to define neoantigen in lung adenocarcinomas, we utilized whole exome sequencing (WES) to determine the mutations that originated single nucleotide variants (SNV) and insert/deletion mutations (InDel), as well as mutated peptides with different antigenicity profiles. Tumor and paired adjacent non-tumor tissues were collected from four patients with lung adenocarcinoma (LUAD) (**Table 1**). Given that there exists progressive genomic evolution from pre-invasive adenocarcinomas to invasive adenocarcinomas, we collected four tissues, including two pre-invasive foci and two invasive foci from the same patient with multifocal carcinomas (patient 4). We collected seven pairs of lung adenocarcinomas and adjacent tissues, and identified a total of 1514 somatic mutations in 1016 genes (excluding silent variants) (**Figures 1A, B**). Among the mutations involving changes in nucleotides, the majority were missense mutations (75.4%), and less common mutations were frame-shift-deletions (8.8%), in-frame-deletions (5.6%) and frame-shift-inserts (3.3%) (**Figure 1A**). Of note, there were little difference in mutated gene numbers between pre-invasive and invasive adenocarcinomas from the same patient (P4.2/P4.4 vs P4.1/P4.3), while higher level of difference was observed in the different tissues within invasive adenocarcinomas (**Figure 1B**). Most of identified mutations were SNVs from missense mutation, but deletions (Del) and insertions (Ins) were also identified. The distribution of variant types displayed similar pattern in pre-invasive and invasive adenocarcinomas as well as in different individuals (**Figure 1C**). The predominant class of SNV, which accounted for the most proportion of the variant types, was found to be C > T and T > C, which means the DNA substitution mutations were most commonly transitions. It appeared that there were no significant transforms of variant types and SNV classes from non-invasive to invasive adenocarcinomas (**Figure 1D**), suggesting their similar mutation pattern.

Then, we utilized the oncoplot to identify top 100 mutated genes ranked by decreasing frequency, including Mucin 16 (MUC16), epidermal growth factor receptor (EGFR) and RNA−binding motif protein 10 (RBM10) that had been previously reported as recurrently mutated in LUAD. The distribution oncoplot of mutated genes also revealed no significant difference between pre-invasive or invasive carcinoma (**Figure 1E**). In addition, three genes (BCLAF1, USP6 and RPL19) sharing the same type of mutation were identified across all 4 patients. Further, the higher expression of 98 genes (involved in TCGA dataset) among 100 mutated genes in above seven pairs of tissues were found to have an association with worse outcome in LUAD, which meant the mutation of these genes may be potential targets for neoantigen screening and therefore improve the immunogenicity of cancer tissues (**Figure 1F**).

### 3.2 The levels of Tumor neoantigen burden and tumor mutational burden across individuals with LUAD and between invasive and non-invasive foci

**Figure 2:**
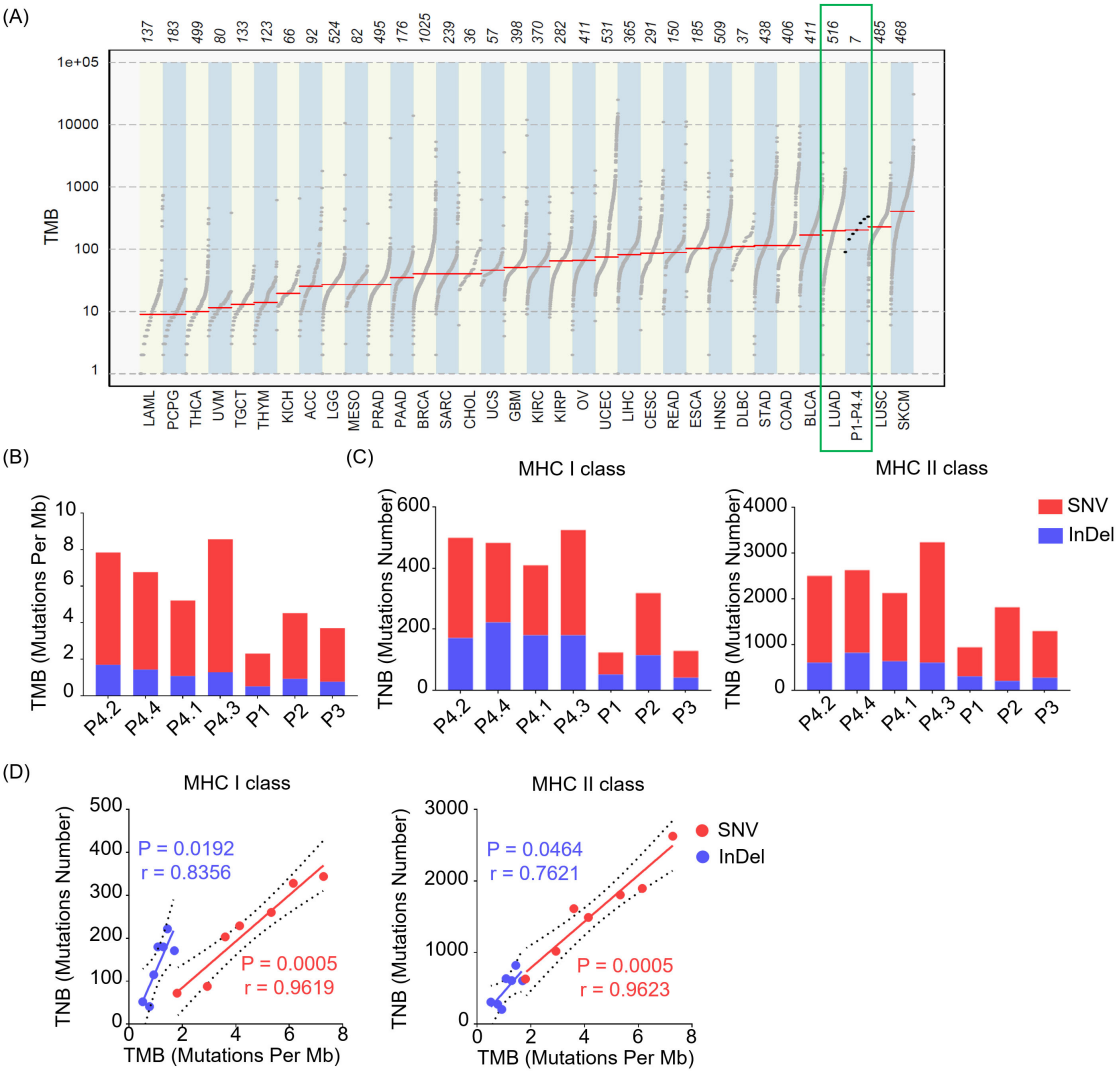
The levels of TMB and TNB in LUAD across different patients and between non-invasive and invasive foci. **(A)** Tumor mutational burden (TMB) profiles over 30 different cancer types from The Cancer Genome Atlas (TCGA) database and our samples (P1- P4.4) in this study. **(B)** Bar plot depicts TMB across individuals of our samples (P1- P4.4) derived from SNVs and InDels. **(C)** Bar plot shows the levels of tumor neoantigen burden (TNB) presented by MHC class I (MHC I, left) or MHC class II (MHC II, right) molecules derived from SNVs and InDels. **(D)** Relationship between TMB and TNB presented by MHC class I (MHC I, left) or MHC class II (MHC II, right) molecules. The green squares represent the TMB profiles of tissues from LUAD and our samples. SNV (Red), InDel (Blue).

An important predictive biomarker for immunotherapy response that garnered significant attention is tumor mutational burden (TMB), which could reflect the number of somatic mutations per megabase of interrogated genomic sequence objectively. The higher of TMB observed, the more chances that the tumor would be explored to trigger T cell responses. To assess the TMB level of the LUAD, we utilized the ‘mutload’ function from the ‘maftools’ package in the R software to generated TMB profiles over 30 different cancer types from The Cancer Genome Atlas (TCGA) database. The results unveiled that the TMB levels of LUAD surpassed those in the majority of other cancer types, indicating a more favorable prospect for immunotherapy in the case of LUAD. In addition, the seven LUAD samples included in this study exhibited similarly high TMB levels as the average values observed in TCGA’s LUAD dataset, suggesting their representativeness for LUAD samples (**Figure 2A**). We next compared TMB levels introduced by SNVs causing single acid amino mutation and InDels changing reading frame on the protein across individuals and between invasive and non-invasive foci. There was minimal difference in TMB levels among between pre-invasive and invasive adenocarcinomas tissues, while higher diversity in TMB levels was observed among individual patients (**Figure 2B**).

Tumor neoantigen burden (TNB) quantifies the neoantigens generated by somatic mutations in tumor cells, serving as a valuable predictor for the efficacy of immunotherapy in cancer. A higher TNB provides a greater pool of potential neopeptides that can be presented by MHC class I (MHC I) or MHC class II (MHC II) molecules, which is crucial for designing personalized precision therapeutic strategies. Here, we observed that both SNVs and InDels contributed to TNB of mutated peptides restricted by MHC I or MHC II molecules (**Figure 2C**) across individuals, with SNVs predominantly driving the contribution. Of note, there was minimal difference in TNB levels between pre-invasive and invasive adenocarcinomas tissues. Although not all mutations contribute to the generation of neoantigen, the higher TMB correlates with a likelihood of having a significant TNB. Consistently, we observed a positive correlation between TNB and TMB, with a higher correlation index in SNV group compared to InDel group (**Figure 2D**).

### 3.3 Identification of neopeptide candidates with high binding affinity to MHC molecules

Given that MHC I presentation is crucial for activating cytotoxic T lymphocytes (CTLs) to direct killing tumor cells, we next performed MHC I-associated neopeptides screening. Among the four enrolled patients, patient 4 possessed HLA-A*0201 allele, which is most frequently observed HLA allele in global population. In addition, patient 4 was diagnosed as multifocal LUAD with sampling of various tumor foci that was interpreted as distinct primary tumors.

**Figure 3:**
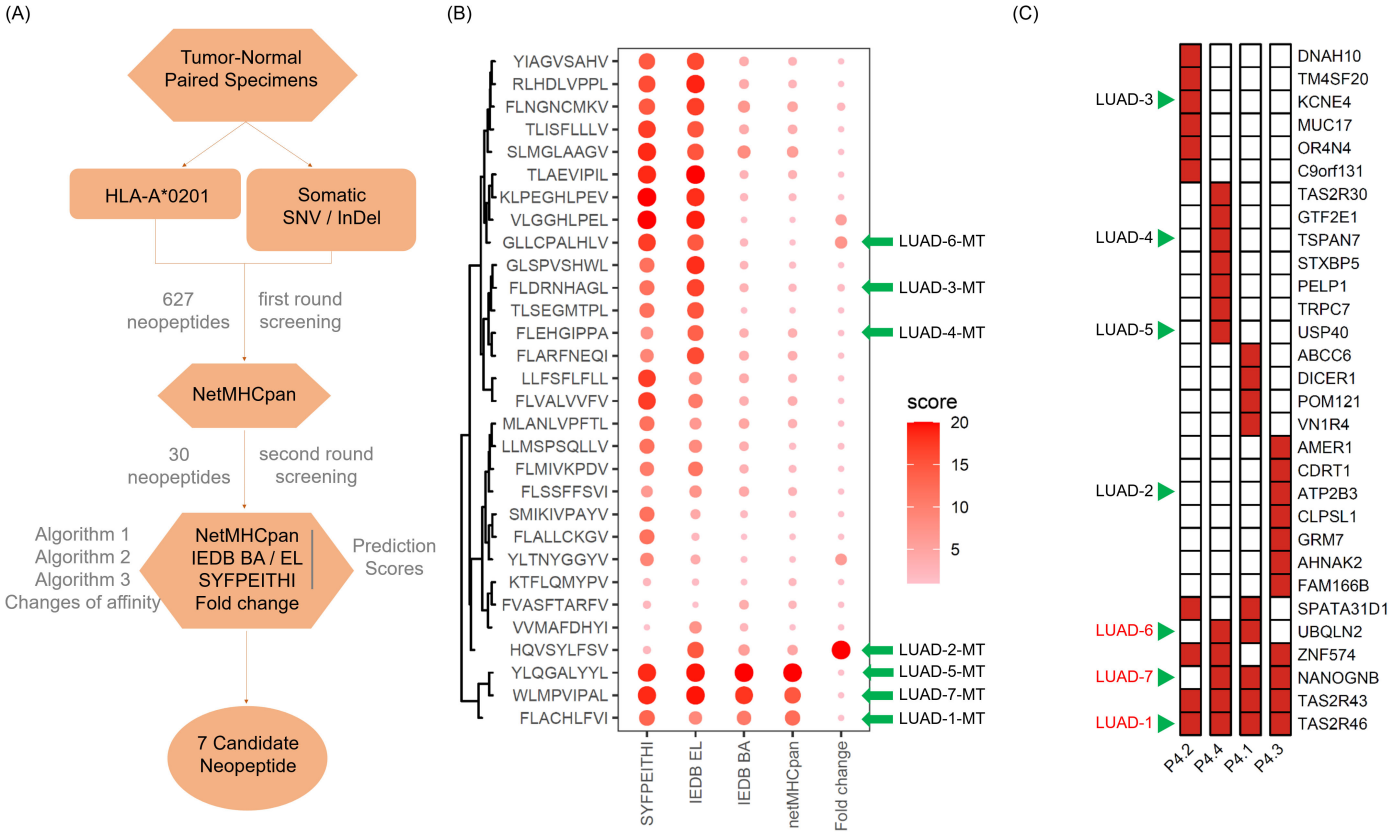
Screening and feature identification of neopeptide candidates within a multifocal LUAD patient with HLA-A*0201 allele. **(A)** Pipeline for neopeptide candidates screening from tumor foci of patient 4. **(B)** Dot plot depicts affinity scores of top 30 mutated peptides with highest predicted HLA-A*0201-binding affinity in the first-round screening. Larger and darker dots represent higher affinity scores or potential immunogenicity. **(C)** Heatmap displays the distribution of genes of the 30 mutated peptides. The green arrows indicate the selected gene candidates and derived neopeptides.

Through the somatic mutation dataset of patient 4, we firstly identified the top thirty HLA-A*0201-restricted neopeptides ranked by peptide-MHC binding affinity scores by NetMHCpan algorithm and identified 30 neopeptides with highest HLA-A2 binding affinity, which already removed repeated genes or peptides (**Figures 3A, B**). In order to mitigate the potential for bias resulting from the use of a single method and eliminate deviations, we conducted an additional screening round using four distinct algorithms: NetMHCpan, IEDB EL, IEDB BA, SYFPEITHI (**Figures 3A, B**). In addition, fold change values reflecting affinity changes of mutated peptides versus wild-type peptides were also calculated (**Figures 3A, B**). Taking into account the binding affinity predicted by multiple algorithms, along with the assessment of synthesis difficulty and dissolution rate, seven neopeptide candidates (ranging from LUAD-1-MT to LUAD-7-MT) were selected from the top 30 neopeptides exhibiting highest HLA-A2 binding affinity for the *in vitro* immunogenicity validation experiment (**Figure 3B**). The basic information, including gene symbol, peptide sequence, mutation type and tissues distribution, of seven neopeptide candidates and paired wild-type peptides (ranging from LUAD-1 to LUAD-7) was identified (**Table 2**). The distribution of top 30 genes, including seven gene candidates, in patient 4 between pre-invasive and invasive carcinomas was displayed, showing that three candidates from UBQLN2, NANOGNB and TAS2R46 genes were distributed in at least 2 out of 4 foci in the patient 4 (**Figure 3C**). Of note, theses three genes were also included in the list of the top 100 mutant genes.

**Table 2:** Basic information of neopeptide candidates.

Gene ID	Gene symbol	Peptide sequence		Mutation type	Tissues distribution
		wild-type (WT)	mutation (MT)		
LUAD-1	TAS2R46	FLVCHLFVI	FLACHLFVI	SNV^a^	P4.1,P4.2,P4.3, P4.4
LUAD-2	ATP2B3	TKSATSSVF	HQVSYLFSV	InDel^b^	P4.3
LUAD-3	KCNE4	GIFLIGIML	FLDRNHAGL	InDel	P4.2
LUAD-4	TSPAN7	FLEHGIPPS	FLEHGIPPA	SNV	P4.4
LUAD-5	USP40	YLQGAPYYL	YLQGALYYL	SNV	P4.4
LUAD-6	UBQLN2	GPTVSSAAPS	GLLCPALHLV	InDel	P4.1,P4.4
LUAD-7	NANOGNB	WLTPVIPAL	WLMPVIPAL	SNV	P4.1,P4.3, P4.4

a SNV, Single nucleotide variants.

b InDel, Insert/deletion mutations.

### 3.4 Proteins encompassing neoantigen candidates are readily detectable in LUAD

**Figure 4:**
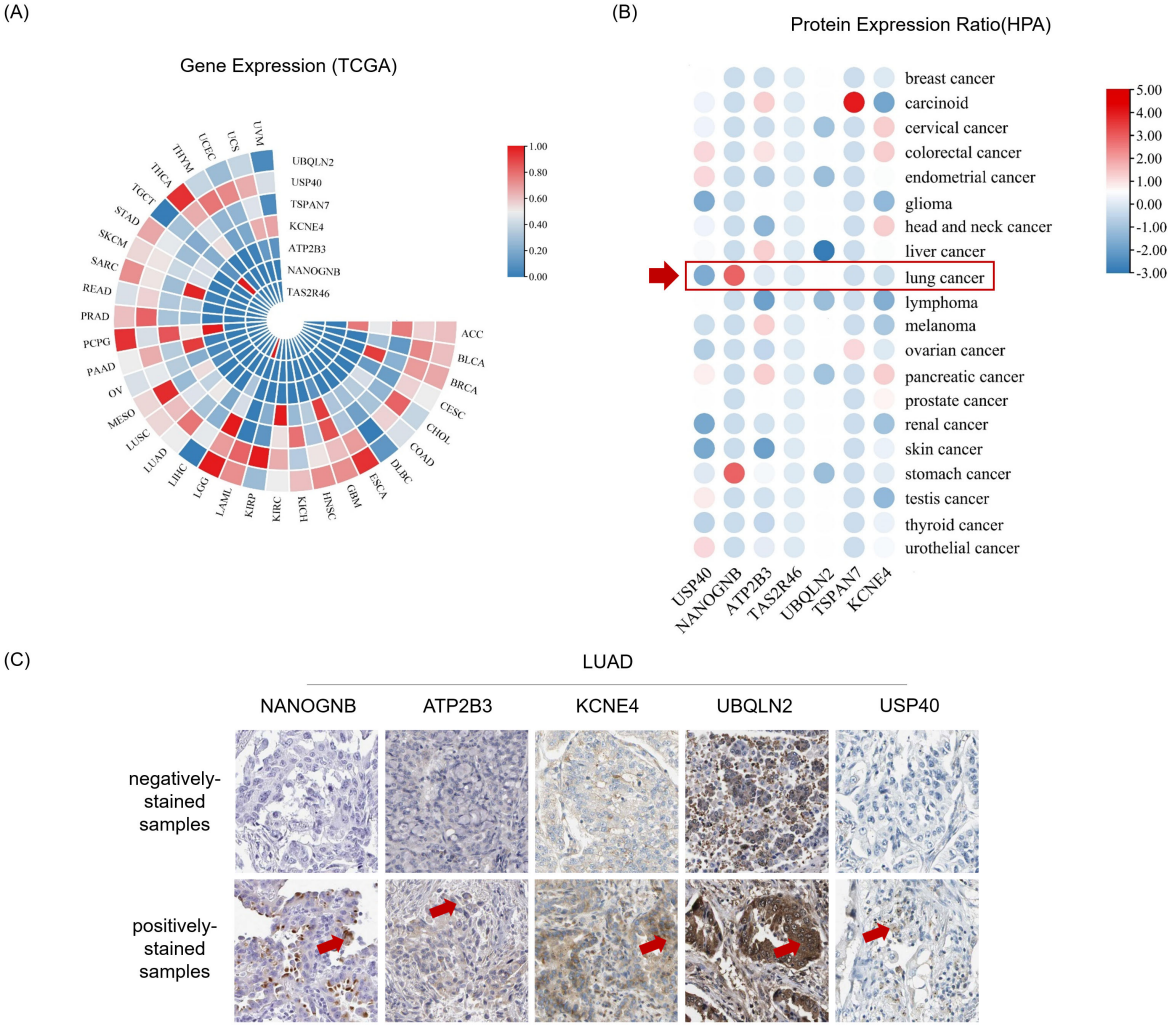
Genes and proteins encompassing neopeptide candidates are detectable in LUAD. **(A)** Heatmap depicts gene expression in pan-cancer tissues from The Cancer Genome Atlas Program (TCGA) datasets. **(B)** Heatmap depicts protein expression ratio (frequency of positive detection in the tissues tested by the corresponding protein) in pan-cancer tissues from The Human Protein Atlas (HPA) database. The red arrow indicates for lung cancer tissues. **(C)** The immunohistochemistry images show representative samples with indicated antibodies negatively stained (negatively-stained samples) or positively stained (positively-stained samples) from the Human Protein Atlas database. Antibody positively stained area of these tissues was indicated by red arrow.

Before initiating *in vitro* cell culture experiments with peptide candidates, we firstly identified corresponding genes for the top seven peptides with the highest affinity, which included ubiquilin 2 (UBQLN2), ubiquitin specific peptidase 40 (USP40), tetraspanin 7 (TSPAN7), potassium voltage-gated channel subfamily E member 4 (KCNE4), ATPase plasma membrane Ca2+ transporting 3 (ATP2B3), NANOG neighbor homeobox (NANOGNB), and taste 2 receptor member 46 (TAS2R46) (**Table 2**). Next, we searched The Cancer Genome Atlas (TCGA) database for transcript levels as well as the Human Protein Atlas (HPA) database for protein levels of the seven candidates in pan-cancer tissues, especially in LUAD tissues. The results revealed detectable mRNA expression of the seven gene candidates in all thirty-three cancer types, with some genes showing generally higher expressed in specific cancer tissues (**Figure 4A**). After checking the protein expression levels in various cancer tissues using HPA database, we observed that among the seven candidates, NANOGNB was the most frequently expressed genes in lung cancer tissues (**Figure 4B**). Besides NANOGNB, other four genes, including ATP2B3, KCNE4, UBQLN2 and USP40, were also found to be positively expressed in some LUAD tissues (**Figure 4C**), which confirmed the importance of these genes in LUAD.

### 3.5 Artificial antigen presenting cells with both peptide-MHC and costimulatory signals are set up for screening neoantigen

**Figure 5:**
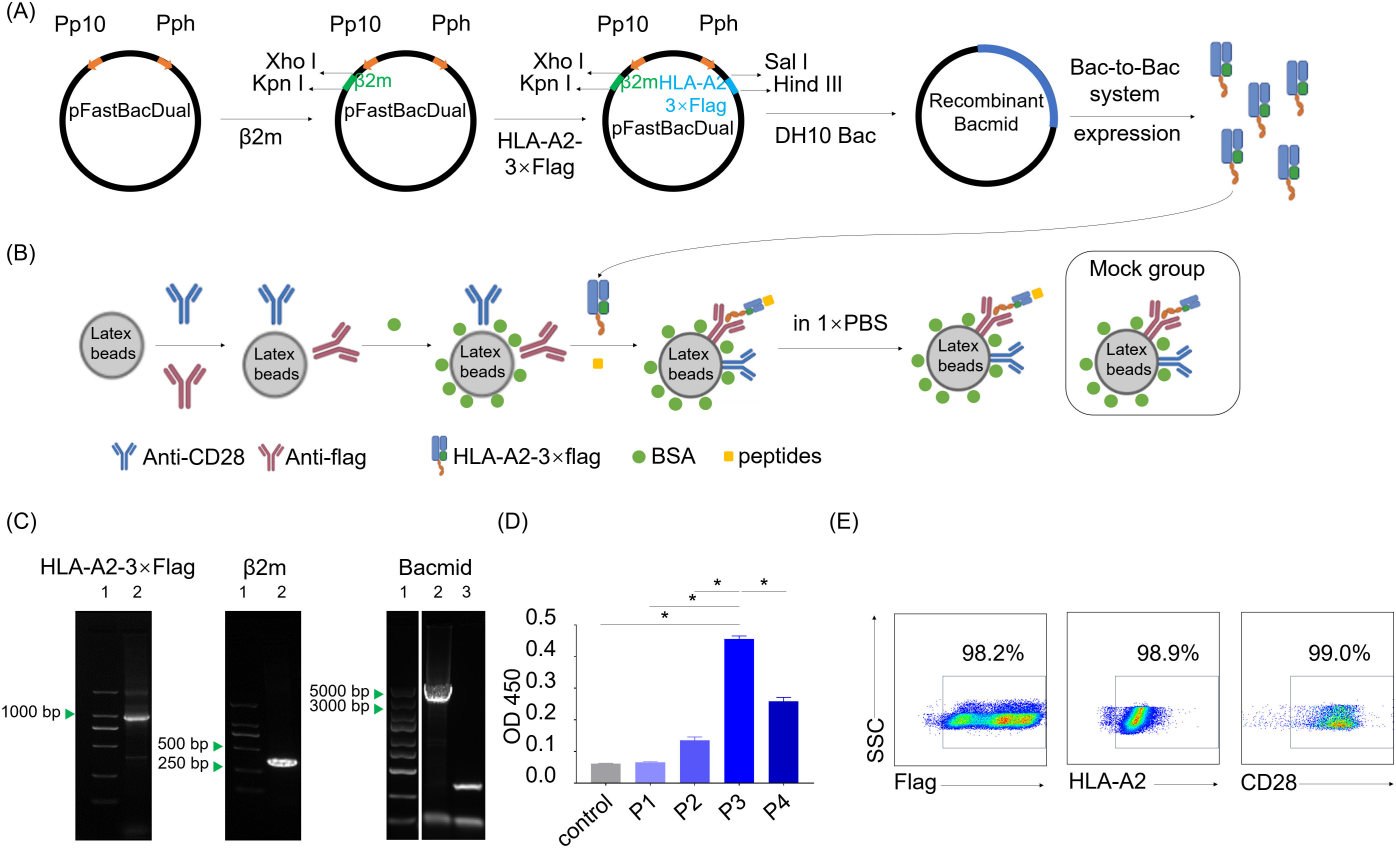
Preparation of dimeric HLA-A*0201 molecules and HLA-A2-laoded artificial antigen presenting cells. **(A)** Schematic shows the construction of recombinant pFastBacDual-β2m-HLA-A2-3×flag-tag plasmid and the following HLA-A2-3×flag protein expression according to Bac-to-Bac baculovirus expression system. **(B)** Schematic shows the construction of HLA-A2 aAPCs with or without (Mock group) peptides pulsed on. **(C)** Agarose gel electropherogram analysis of two genes in the pFastBacDual-β2m-HLA-A2-3×flag-tag plasmid and recombinant bacmid. Left (lane1: marker, lane2: HLA-A2-3×flag), middle (lane1: marker, lane2: β2m), right (lane1: marker, lane2: recombinant bacmid, lane3: control bacmid). **(D)** Detection of the HLA-A2-3×flag protein by sandwich ELISA with W6/32 and HRP-anti-human β2m. Supernatants of the uninfected Spodoptera frugiperda cells (Sf9 cells) (control) and P1, P2, P3, P4 derived from baculovirus-infected Sf9 cells were tested. Data are presented as means ± SEMs. Data were analyzed by Student’s t-test or ANOVA for comparisons of groups. P<0.05 was considered statistically significant. * for P<0.05. **(E)** Scatter plots depict the frequency of flag, HLA-A2 and CD28 on the aAPCs.

To test the immunogenicity of neopeptide candidates, we constructed an artificial antigen presenting cell (aAPC) system to manipulate peptide-MHC signals together with costimulatory signals for T cell activation assay. The recombinant vector pFastBacDual-β2m-HLA-A2-3×flag-tag was constructed to express HLA-A*0201 fusion protein with a 3×Flag tail (HLA-A2-3×flag, **Figure 5A**). Recombinant HLA-A2-3×flag produced by Bac-to-Bac system were loaded on the latex beads together with anti-CD28 antibody providing co-stimulatory signal to prepare HLA-A2 aAPCs. Neopeptide candidates were pulsed onto the HLA-A2 aAPCs to stimulate PBLs. aAPCs pulsed with CMV peptide was regarded as a positive control, and aAPCs without peptide pulsing was regarded as a negative control (Mock group, **Figure 5B**).

PCR, agarose gel electrophoresis and DNA sequencing confirmed the accuracy of recombinant genes encoding HLA-A2-3×flag protein in the recombinant vector pFastBacDual-β2m-HLA-A2-3×flag-tag (**Figure 5C**). P3 viral stock from pFastBacDual-β2m-HLA-A2-3×flag-tag-transfected spodoptera frugiperda cells (Sf9 cell) demonstrated a higher HLA-A2-3×flag concentration than other groups (**Figure 5D**), and thus HLA-A2 aAPCs were prepared with P3 viral stock. Flow cytometry detection revealed HLA-A2 aAPCs properly coated with anti-CD28 antibody and HLA-A2-3×flag proteins (**Figure 5E**). Together, the HLA-A2 aAPCs was successfully set up for the following co-culture step for neopeptides validation.

### 3.6 Candidate LUAD-7-MT encoded by NANOGNB exhibits higher immunogenicity than its wild type counterpart LUAD-7-WT

**Figure 6:**
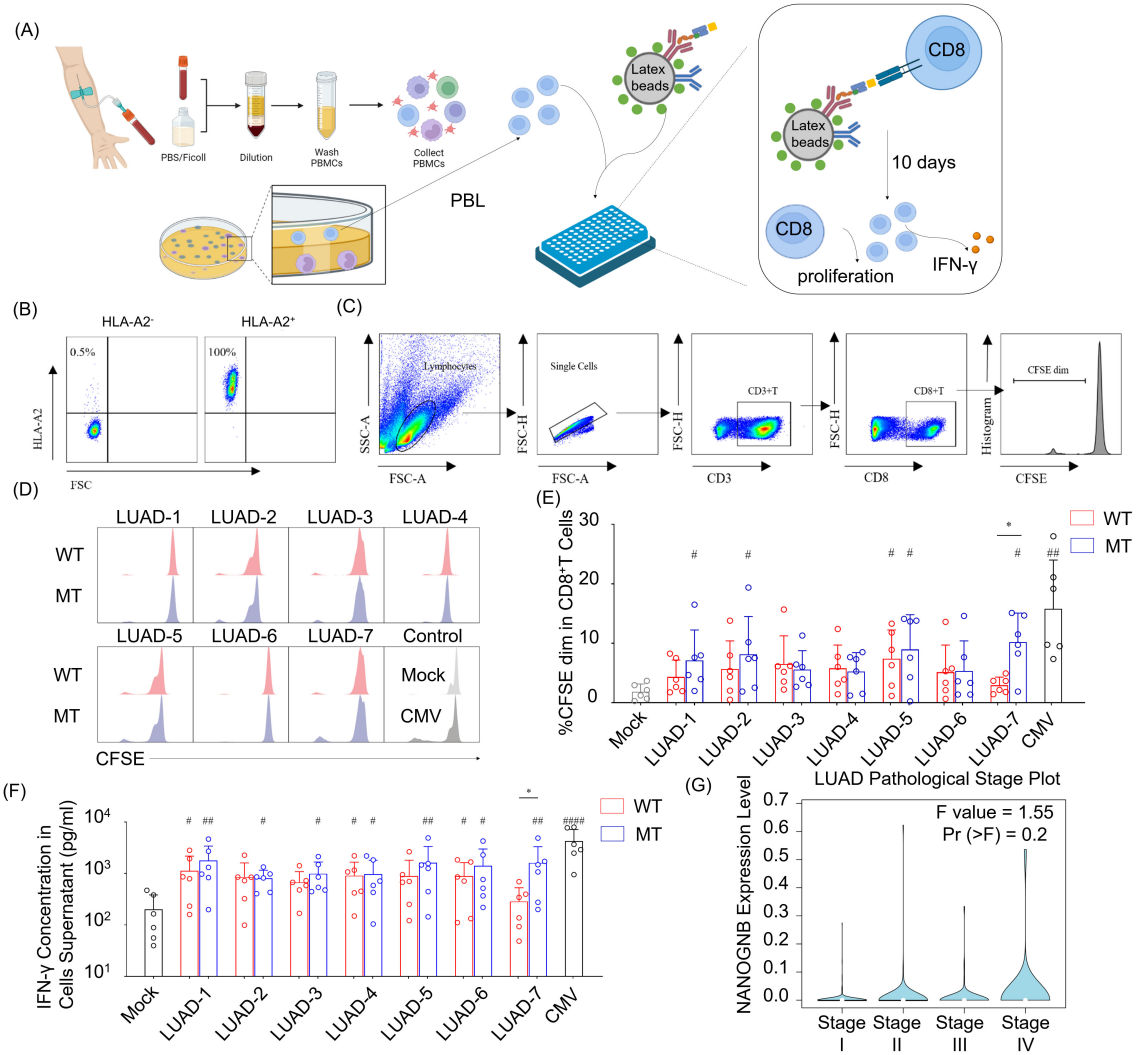
NANOGNB-derived LUAD-7-MT peptide displays elevated immunogenicity compared with wild-type counterpart, promoting heightened cell proliferation and IFN-γ secretion of T cells. **(A)** Flowchart for the immunogenic neoantigen peptides validation procedure. **(B)** Scatter plot of HLA-A2 staining in PBMC. **(C)** Gating strategy for flow cytometry analysis to identify CD3+ CD8+ CFSE dim cell subset. **(D)** Representative histogram graph depict CFSE staining in CD8+ T cells among seven pairs of peptides, negative control (Mock group) and positive control (CMV group). WT group (red), MT group (blue). **(E)** Summary bar graph shows the percentage of the CD3+ CD8+ CFSE dim cell subset in each group. **(F)** Summary bar graph shows the concentration of the IFN-γ in cell supernatants in each group. **(G)** Violin plot shows the expression of NANOGNB in different pathological stages of LUAD. Data are presented as means ± SEMs. Data were analyzed by Student’s t-test or ANOVA for comparisons of groups. P<0.05 was considered statistically significant. * for P<0.05 compared with paired peptide. # for P<0.05 compared with Mock group. ## for P<0.01 compared with Mock group, #### for P<0.0001 compared with Mock group. (Created with BioRender.com).

To assess their immunogenicity, seven neopeptide candidates (LUAD-1-MT to LUAD-7-MT) were individually pulsed onto HLA-A2 aAPCs, with corresponding wild type peptides (LUAD-1-WT to LUAD-7-WT) serving as controls. The peptide-pulsed HLA-A2 aAPCs were co-cultured with peripheral blood lymphocytes (PBLs) with a ratio of 1:5 (aAPCs: PBLs) for ten days. The peptides, presented by HLA-A2, provided TCR signaling of CD8+ T cells, and anti-human CD28 on the aAPC provided a costimulatory signal. After ten days coculturing, supernatant and activated PBLs were collected for IFN-γ detecting and CD8+ T cells proliferation, respectively (**Figure 6A**).

PBLs were gathered from healthy individuals positive for HLA-A2 allele to ensure compatibility with the HLA-A2 on aAPCs (**Figure 6B**). The percentage of cells exhibiting diluted 5-(and 6)-carboxyfluorescein diacetate succinimidyl ester (CFSE) indicated that the initiation of CD8+ T cells proliferation occurred with CMV- and several candidate peptides-pulsed aAPCs (**Figures 6C–E**). Of note, only the LUAD-7-MT peptide exhibited a higher capability to promote CD8+ T cells proliferation compared to the wild type control LUAD-7-WT. In addition, aAPCs pulsed with LUAD-7-MT induced greater IFN-γ secretion compared to those pulsed with LUAD-7-WT-pulsed aAPCs (**Figure 6F**). These results suggested that the LUAD-7-MT neopeptide may outperform the other six neopeptides, making it a potential novel target for T cell-based immunotherapy in LUAD, particular for patient 4. Given that the immunogenic LUAD-7 peptide originates from NANOGNB, we next assessed the average expression of NANOGNB in LUAD at different disease stages. Transcript levels of NANOGNB increased with the progression of LUAD (**Figure 6G**). This suggested that NANOGNB could be a promising target for immunotherapy, particularly in advanced stages of LUAD.

## 4 Discussion

In this study, we present the mutation spectrum of a cohort of lung adenocarcinoma (LUAD) tissues. Despite previous studies demonstrating distinct mutational features between pre-invasive and invasive adenocarcinomas (30, 31), our results do not exhibit obvious variances in tumor mutation burden (TMB), neoantigen burden (TNB), or gene mutation. In addition, our comprehensive screening system, integrating WES analysis, MHC-peptide affinity prediction, and *in vitro* T cell stimulation, has conclusively revealed that the potential of the neopeptide LUAD-7-MT. Originating from the NANOGNB gene, this peptide stands out as a promising target for T-cell based immunotherapy in LUAD.

Due to the limited immunogenicity of peptides derived from tumor antigens, identifying tumor neopeptides has emerged as a strategy to enhance tumor immunogenicity and evoke T cell immune responses (32, 33). Recently, several methods, including NeoScreen and trogocytosis reporting system, have been set up to detect the immunogenicity of neopeptides. In the context of Neoscreen, engineered B cells are set up as antigen-presenting cells, by co-electroporating RNA encoding the immune stimulatory 4-1BB ligand, OX40 ligand, and IL-12. These engineered B cells acquire CD40 activity, effectively stimulating neoepitope-specific CD8+ tumor-infiltrating lymphocytes (TILs) *ex vivo* (34). In another system, target antigen library is transduced into host cells equipped with a trogocytosis reporting system. These cells are then co-cultured with T cells possessing TCRs, following by detection of highly specific nibbling process, occurring exclusively between cells recognized by the TCR-peptide-MHC complex successfully (35, 36). It is noteworthy that both two methods require a substantial investment in terms of manpower and material resources. Here, we have established a tumor neopeptide screening and validation system through an *in vitro* experiment based on artificial antigen-presenting cells (aAPCs), which present neopeptide candidates to CD8+ T cells. Unlike the complex and challenging preparation of engineered B cells or trogocytosis reporting host cells, a notable advantage of our aAPCs is their convenient preparation in a ready-to-use format. The prepared aAPCs can be transported and stored for extended periods while maintaining stability. This stability is a crucial factor that facilitate the successful execution of numerous experiments, ensuring repeatability across multiple batches.

Neopeptides, originating from somatic mutations in cancer cells, are unique to each individual’s tumor and play a vital role in shaping the immune response against cancer (37). The clinical significance of neoantigen lies at the forefront of advancing personalized cancer immunotherapy and precision medicine (38, 39). Therapies designed to stimulate the immune system, such as checkpoint inhibitors and adoptive T cell therapies, can be tailored to target neopeptides, promoting a more precise and effective immune response against cancer cells. TNB has shown promise as prognostic biomarkers in cancer (40, 41). Higher neopeptides load is often associated with improved responses to immunotherapy and better clinical outcomes (42). In the context of immune checkpoint inhibitors, tumors with a higher TNB may have a more immunogenic profile, potentially enhancing the effectiveness of checkpoint inhibitor therapies (43). In the present study, the absence of distinct variances in TMB, TNB, or gene mutation was observed between pre-invasive and invasive adenocarcinomas, which suggests that traditional immunotherapeutic approaches relying on checkpoint inhibitors may not exploit significant differences between pre-invasive and invasive stages of adenocarcinoma.

Nowadays, neoantigen vaccines have transitioned into the clinical trial stage and have shown promising clinical treatment potential (16, 44). A study conducted four years post-treatment with a neoantigen vaccine revealed the sustained presence of neoantigen-specific T cells in eight melanoma patients. Remarkably, these T cell clones displayed ongoing diversification, presenting various clonal types (45). Notably, a significant proportion of clinical trials, approximately 75% of clinical trials employ mRNA-, DNA- or peptide-based antigen delivery platforms. Additionally, more than 75% of personalized therapeutic cancer vaccines currently in phase 1–2 are designed to target a total of ten or more neoantigens (46). Overall, cancer neoantigen vaccines may be the next preferred combination partner for long-term cancer treatment, providing a platform that can be easily combined with other existing cancer treatments with minimal biotoxicity and a favorable safety profile (47–49). Meanwhile, personalized neoantigen-based vaccines can be produced to treat individual cancer patients safely and to produce specific T cell responses (14). Regarding to the genes linked to the screened neoantigens in our study, it appears that mutated NANOGNB genes may exhibit heightened immunogenicity, potentially contributing to the activation of CD8+ T cells. This proposition gains support from recent findings indicating that individuals harboring NANOGNB mutations are more prevalent among patients who respond favorably to immune checkpoint inhibitor treatments compared to those with mutations in other responsive-related genes (50). The alignment of these findings across studies reinforces the potential significance of NANOGNB mutations in shaping immune responses and consequently enhancing the prospects of immunotherapy.

We compare the mutated genes between pre-invasive adenocarcinomas and invasive adenocarcinomas in lung cancer and find no significant difference. Thus, we establish a neopeptide screening and validation system to further define immunogenic information from WES. Importantly, we characterized LUAD-7-MT neoantigen peptide as a novel HLA-A2-restricted, immunogenic NANOGNB-derived CTL epitope that can be processed and presented by tumor cells. Taken together, these findings indicate that highly immunogenic LUAD-7-MT neoantigen peptide is potentially useful for the development of clinical treatment target, which is capable of effectively enhancing LUAD-7-MT reactive CTL response in NANOGNB-expressing LUAD patients.

The system described here provides an integrated approach for screening tumor immunogenic neoantigens, which could advance the development of personalized immunotherapy. However, limitations remain, including a small sample size, reliance on a single HLA molecule, absence of peptide/HLA tetramer validation, and lack of data on the expression levels of the seven candidate markers in patient-derived samples or cell lines. Future studies should address these by expanding the sample size with a broader range of HLA molecules, and using tetramer staining to more accurately identify neopeptide-specific T cells. Additionally, confirming immunogenicity through *in vivo* studies would be essential before clinical translation, and further research on the immunogenicity and anti-tumor efficacy of neopeptides in HLA transgenic mice will provide more accurate insights and therapeutic guidance. Given the high heterogeneity of neoantigens across individuals, the absence of antibodies specific to individual mutated antigens limits the use of IHC staining to detect mutated proteins. Developing antibodies against mutated epitopes would help verify the distribution frequency of these mutated proteins within the population, potentially extending applications from personalized therapy to broader, population-based treatments.

## Data Availability

The raw sequence data reported in this paper have been deposited in the Genome Sequence Archive (Genomics, Proteomics & Bioinformatics 2021) in National Genomics Data Center (Nucleic Acids Res 2024), China National Center for Bioinformation / Beijing Institute of Genomics, Chinese Academy of Sciences (GSA-Human: HRA009317) that are publicly accessible at https://ngdc.cncb.ac.cn/gsa-human.
